# Effect of the TrFE Content on the Crystallization and SSA Thermal Fractionation of P(VDF-*co*-TrFE) Copolymers

**DOI:** 10.3390/ijms231810365

**Published:** 2022-09-08

**Authors:** Nicolás María, Florian Le Goupil, Dario Cavallo, Jon Maiz, Alejandro J. Müller

**Affiliations:** 1 POLYMAT and Department of Polymers and Advanced Materials: Physics, Chemistry and Technology, University of the Basque Country UPV/EHU, Paseo Manuel de Lardizábal 3, 20018 Donostia-San Sebastián, Spain; 2 Laboratoire de Chimie des Polymères Organiques (LCPO-UMR 5629), Bordeaux INP, Universitè de Bordeaux, CNRS, 16 Av. Pey-Berland, 33607 Pessac, France; 3 Department of Chemistry and Industrial Chemistry, University of Genova, 16146 Genova, Italy; 4 Centro de Física de Materiales (CFM) (CSIC-UPV/EHU)-Materials Physics Center (MPC), Paseo Manuel de Lardizábal 5, 20018 Donostia-San Sebastián, Spain; 5 IKERBASQUE, Basque Foundation for Science, Plaza Euskadi 5, 48009 Bilbao, Spain

**Keywords:** P(VDF-*co*-TrFE), crystallization kinetics, self-nucleation, SSA fractionation, comonomer inclusion

## Abstract

In this contribution, we study the effect of trifluoro ethylene (TrFE) comonomer content (samples with 80/20, 75/25, and 70/30 VDF/TrFE molar ratios were used) on the crystallization in P(VDF-*co*-TrFE) in comparison with a PVDF (Poly(vinylidene fluoride)) homopolymer. Employing Polarized Light Optical Microscopy (PLOM), the growth rates of spherulites or axialites were determined. Differential Scanning Calorimetry (DSC) was used to determine overall crystallization rates, self-nucleation, and Successive Self-nucleation and Annealing (SSA) thermal fractionation. The ferroelectric character of the samples was explored by polarization measurements. The results indicate that TrFE inclusion can limit the overall crystallization of the copolymer samples, especially for the ones with 20 and 25% TrFE. Self-nucleation measurements in PVDF indicate that the homopolymer can be self-nucleated, exhibiting the classic three *Domains*. However, the increased nucleation capacity in the copolymers provokes the absence of the self-nucleation *Domain II*. The PVDF displays a monomodal distribution of thermal fractions after SSA, but the P(VDF-*co*-TrFE) copolymers do not experience thermal fractionation, apparently due to TrFE incorporation in the PVDF crystals. Finally, the maximum and remnant polarization increases with increasing TrFE content up to a maximum of 25% TrFE content, after which it starts to decrease due to the lower dipole moment of the TrFE defect inclusion within the PVDF crystals.

## 1. Introduction

Nowadays, research on materials composed of polyvinylidene fluoride (PVDF) attracts high interest in the industrial and academic sectors due to its outstanding properties and possible applications in different fields [1,2,3]. This semicrystalline polymer can substitute some inorganic materials with better yield and reduced cost, and at the same time, it can also improve the physical properties of new electronic devices [4,5,6]. PVDF has excellent properties such as good flexibility, low cost, high chemical resistance, and good compatibility with other materials. Moreover, the most interesting properties of PVDF are ferroelectricity and piezoelectricity because of the use of PVDF, e.g., in sensors [7,8], capacitors [9], and even in renewable energies [10,11]. Another essential characteristic of PVDF is polymorphism. The polymer can crystallize in at least four different phases (α, β, γ, and δ-phase) [12,13]. The most stable crystalline phase, obtained when crystallization occurs from the melt, is the α-phase, but this is a nonpolar phase (paraelectric) that is useless in the previously mentioned electronic applications [14]. The rest of the crystalline phases are ferroelectric and piezoelectric at different levels, β- being the phase with the highest polarization due to its all-*trans* chain conformation [15,16]. In bulk PVDF, the crystallization process of all these polar phases is complex, and numerous studies have been published in the literature during the last few years. In PVDF films or fibers, mechanical stresses, such as stretching, are good methods to obtain PVDF β-phase due to a transition that occurs from α- to β-phase in the solid state [17,18]. Moreover, the addition of several salts, such as Mg(NO_3_)2-6H_2_O, to PVDF solutions and the posterior preparation of thin films can also promote the formation of the β-polar phase [19].

One of the best options to increase PVDF polarization is to copolymerize with trifluoroethylene (TrFE) [20]. These random copolymers always crystallize in the all-*trans* chain conformation when crystallization occurs from the melt. Apart from this advantage, P(VDF-*co*-TrFE) copolymers also have good flexibility. They are biocompatible and can be used as sensors in biological environments [21]. These properties make these copolymers very interesting for biosensors and medical applications [22,23]. The behavior in the crystallization process of the PVDF component within the copolymer depends on the composition, and it is well-known that P(VDF_75_-*co*-TrFE_25_) has the highest ferroelectric response in terms of polarization, coercive field, and switching rate [24]. In addition, another important parameter involved in these copolymers, which depends on the composition, is the Curie temperature. Lovinger et al. demonstrated by X-ray experiments that the Curie transition depends on the amount of PVDF in the copolymers, ranging from 52 to 78% [25]. When the amount of PVDF increases, the Curie transition also increases, up to 80% of PVDF, where the transition almost merges with the melting temperature. Interpolating the data obtained at different compositions, they estimated that the Curie temperature for the PVDF homopolymer is around 205 °C, i.e., above its melting temperature. A recent study published by Meereboer et al. demonstrated that confining P(VDF-*co*-TrFE) in nonpolar matrix results in a slight increase in the Curie transition. However, when a more polar matrix is used, the Curie transition temperature is drastically reduced due to the crystallite size reduction [26].

Piezoelectric properties are also an essential point for P(VDF-*co*-TrFE) copolymers. During the last years, thin films (~1 μm) of P(VDF-*co*-TrFE) have been studied to employ them as pressure sensors in a wide range of pressures (0–300 mmHg) with fast recovering times (0.17 s) and with high all-trans conversion [22]. Moreover, Zhaoyang et al. [27] employed this type of thin film as nanogenerators and exhibited reasonable electrical outputs and good stability. The spin coating technique is employed to prepare all thin films presented in these works. These nanogenerators have the capacity to convert mechanical energy into electrical energy in flexible substrates. Finally, P(VDF-*co*-TrFE) thin films can also be applied for energy harvesting applications in microelectromechanical systems (MEMS) [28]. The excellent piezoelectric, ferroelectric, and dielectric response exhibited by this copolymer has made P(VDF-*co*-TrFE) a very appropriate material for the applications described above.

Up to now, in several works, crystal characteristics, structure, and phase transitions of P(VDF-*co*-TrFE) copolymers have been widely studied by X-ray and Raman techniques [29,30,31]. Moreover, the polarization hysteresis loops of P(VDF-*co*-TrFE) thin films have also been analyzed [32,33,34]. Linked with all these properties, processing conditions are another important aspect, and different works discussing their effect are also found in the literature. Annealing processes have been applied at different temperatures, such as 120, 130, or 140 °C, during different times (from 1 to 24 h) to observe how these conditions affect the final structure and its ferroelectric properties [35,36,37]. Spampinato et al. revealed in their work that the annealing temperature affects the remnant polarization value, and they established that the best temperature range for processing was between 133 and 137 °C. Regarding the annealing time, they concluded that only 15 min was enough to obtain high ferroelectric performance and that this annealing time will mainly affect the coercive field value [38].

In this work, we studied the overall crystallization kinetics in P(VDF-*co*-TrFE) random copolymers with different compositions and compare them with a standard PVDF homopolymer to observe how the TrFE comonomer affects crystallization. We employed different experimental techniques such as Differential Scanning Calorimetry (DSC), Polarized Light Optical Microscopy (PLOM), and Wide Angle X-ray Scattering (WAXS). Isothermal and non-isothermal experiments were performed, and the nucleation rate, growth rate, and different kinetic parameters were calculated to determine the nucleating effect of TrFE in PVDF and how this comonomer can affect the crystallization of the all-trans crystalline phase. Additionally, self-nucleation and Successive Self-Nucleation and Annealing (SSA) thermal fractionation studies were performed to investigate the inclusion of TrFE in PVDF crystals. Finally, a full ferroelectric study was performed by recording the polarization versus electric field hysteresis loops of different composition P(VDF-*co*-TrFE)-based capacitors. The results were analyzed and correlated with the kinetics studied by DSC experiments.

## 2. Results and Discussion

### 2.1. Non-Isothermal Crystallization

First, P(VDF-*co*-TrFE) copolymers and the PVDF homopolymer were analyzed by DSC under non-isothermal conditions.

**Figure 1:**
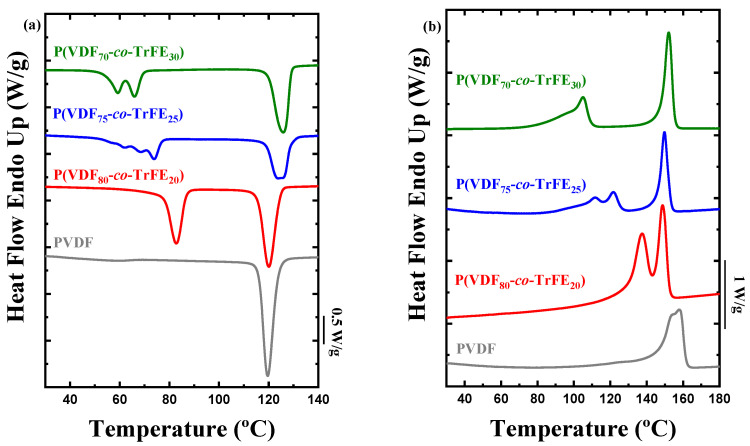
DSC experiments for the PVDF homopolymer and P(VDF-*co*-TrFE) copolymers at different compositions. (**a**) Cooling scan from the melt at 20 °C/min and (**b**) heating scan at 20 °C/min after the previous cooling process.

Figure 1a shows the cooling process from the melt for copolymers and for the neat PVDF. In the homopolymer, only one crystallization peak was observed at 120 °C, whereas in the random copolymers, different exotherms were appreciated. The peak observed at high temperatures (~120–125 °C) for the copolymers corresponds to the crystallization peak of the PVDF α-phase. It can be observed how this crystallization temperature increases when the amount of TrFE also increases. Moreover, in the copolymers, other peaks were appreciated at lower temperatures. These peaks correspond to the PVDF Curie transition, associated with the Curie temperature (*T_Curie_*). This temperature indicates the phase transition between ferroelectricity and paraelectricity of the compounds. At temperatures above this *T_Curie_*, the material is paraelectric, whereas if the system is below the *T_Curie_*, the material is ferroelectric. It is well-known in the literature that for P(VDF-*co*-TrFE) copolymers, when the amount of vinylidenfluroide counits increases, the *T_Curie_* also increases [25,30].

In Figure 1b, the heating DSC curves of the same samples are shown. In this case, the melting peak that corresponds to the PVDF homopolymer is at higher temperatures than the melting peaks observed for the PVDF phase within the copolymers, which appear at temperatures below that of neat PVDF and also below the melting of neat poly(trifluoroethylene) (PTrFE) studied in the literature [39,40]. Similar to the crystallization temperature when the composition of TrFE increases, the melting temperature also increases in the copolymers [41,42]. One hypothesis for this behavior is that it is due to the nucleation effect observed on the PVDF (discussed below), where the TrFE comonomer acts as a nucleating agent, increasing both the crystallization and the melting temperature (only one or two degrees for the melting transition).

For the copolymers, the Curie transition was observed at lower temperatures, below the crystallization exotherm of the PVDF component. This transition exhibits a reversible Curie point at which the ferroelectric polymers show a transformation from a polar ferroelectric state to a nonpolar paraelectric state or vice versa. In the DSC heating scans (Figure 1b), a transition from a ferroelectric to a paraelectric phase appeared [43]. Below this Curie point, the crystalline structure in the ferroelectric phase was composed of all-trans chains (*TTT*). On the other hand, above the Curie point, the paraelectric crystalline structure essentially consisted of a statistical combination of *TT*, *TG^+^*, and *TG^−^* rotational isomers, composed of the α-phase (*TG^+^TG^−^*) and a phase that consists of α-phase with trans defects [37,44]. The WAXS analysis performed at room temperature after the first heating and cooling process (Figure S1) revealed that copolymers crystallize in all-trans conformation (β-phase) and the neat PVDF in the α-phase. Figure S1 shows, for the random copolymers, a shift to lower *q*-values in the reflection of the β-phase when the content of TrFE increase. This shift is generated by the inclusion of the TrFE in the crystals of PVDF [30,42]. All the calorimetric data extracted from the non-isothermal crystallization experiments are listed in Table 1. In this Table, the values of the melting and crystallization enthalpies are presented. In the case of an exclusion of the TrFE in the PVDF crystals, the values of the enthalpies should decrease dramatically when the content of TrFE increases. In our case, this trend does not occur; therefore, this is further evidence of the inclusion of TrFE in the crystals of PVDF.

### 2.2. Isothermal Crystallization

The isothermal crystallization of the PVDF homopolymer and the random copolymers was also studied to determine the kinetics of the crystallization process at different contents of TrFE. First, all the samples were observed on the polarized light optical microscope, and the growth rate of the crystals was measured.

**Figure 2:**
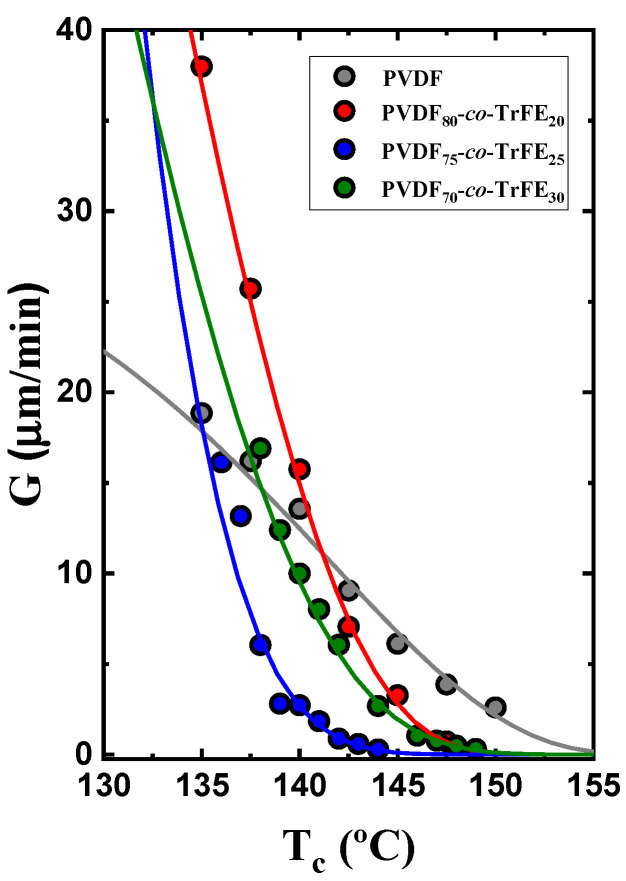
Superstructural growth rates obtained by PLOM for the PVDF homopolymer and P(VDF-*co*-TrFE) random copolymers at different compositions against the crystallization temperature.

Figure 2 shows the isothermal superstructural growth rates (either spherulites or axialites) from the melt of the samples, obtained employing the PLOM technique, where the solid lines plotted are calculated using the Lauritzen and Hoffman theory [45]. In the case of random copolymers, due to the high nucleation density observed, only the crystal growth at crystallization temperatures higher than 135 °C was measured. Figure 2 shows the growth rates (*G*) as a function of the crystallization temperature (*T_c_*). It is observed how neat PVDF superstructures have very different temperature dependence on their growth rates. Hence, the *G* values are faster than the random copolymers at high crystallization temperatures, but the *G* versus *T_c_* curves crossed at lower temperatures. Among the random copolymers, the general trend is that of a reduction in growth rate as TrFE is incorporated in the copolymers, a trend that can be rationalized by the inclusion of TrFE chains within the PVDF crystals, which apparently limit the secondary nucleation process of PVDF chains.

The parameters obtained using the Lauritzen and Hoffman theory are explained and listed in Table S1 of the Supporting Information section.

**Figure 3:**
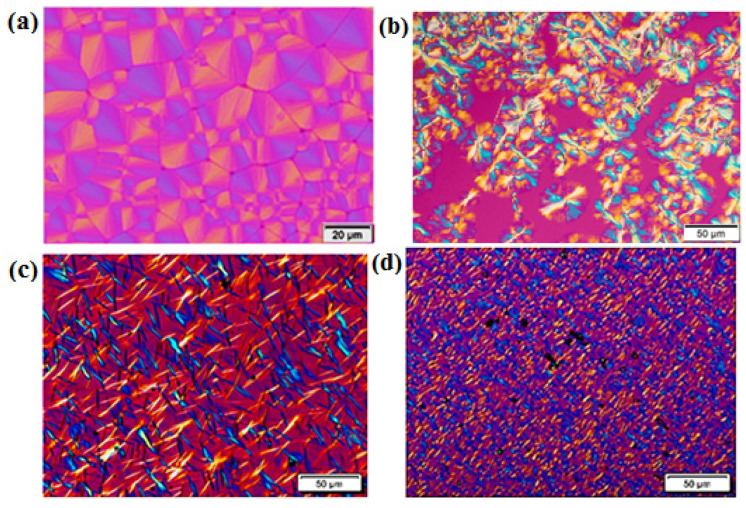
Representative PLOM images taken during an isothermal crystallization process of: (**a**) PVDF homopolymer at 140 °C, (**b**) P(VDF_80_-*co*-TrFE_20_) at 140 °C, (**c**) P(VDF_75_-*co*-TrFE_25_) at 139 °C, and (**d**) P(VDF_70_-*co*-TrFE_30_) at 140 °C.

Apart from the spherulite growth rate, the morphology of the superstructures formed was also studied. As observed in Figure 3a, the PVDF homopolymer exhibits clear negative spherulites with well-defined Maltese cross extinction patterns. As TrFE is incorporated into the copolymers, the morphology changes from spherulites to axialites. This is seen in Figure 3b–d. In Figure 3b, when the TrFE content is still low (80/20), some spherulites are still visible, coexisting with axialites. If the TrFE content increases, the morphology changes to mostly axialites with a relatively similar size (i.e., instantaneously nucleated). In the case of the sample with the highest composition in TrFE, P(VDF_70_-*co*-TrFE_30_) (Figure 3d), the morphology is made of microaxialites where the nucleation density is very high. Figure 3 clearly shows that the inclusion of TrFE has a nucleating influence on PVDF at the examined isothermal crystallization temperatures (indicated in the figure caption), as the number of primary nuclei and its density increases when the amount of TrFE increases in the copolymer.

The crystallization process was also studied by Differential Scanning Calorimetry (DSC) to estimate the primary nucleation rate before crystallization starts (from incubation time data), the overall crystallization kinetics (including both primary and secondary nucleation data), and the melting point of the isothermally crystallized polymorphs.

**Figure 4:**
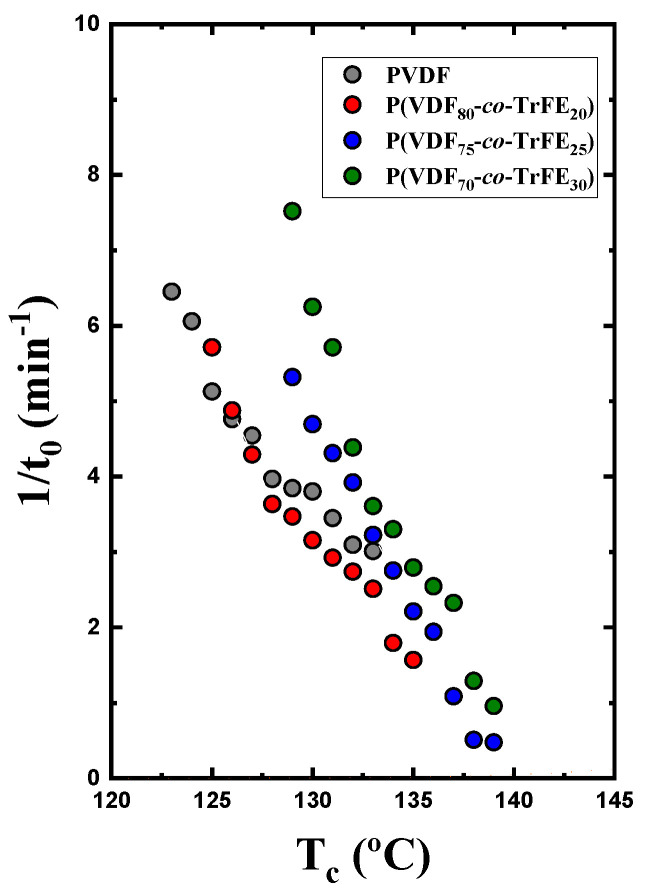
Inverse of the induction time for neat PVDF and copolymers at different compositions against the isothermal crystallization temperature.

The primary nucleation rate was obtained through the inverse of the induction or incubation time (*t*_0_). This represents the primary nucleation rate before any exothermic crystallization heat can be detected in the DSC. Figure 4 shows the inverse of the induction time against the crystallization temperature. At high crystallization temperatures, the samples have similar nucleation rate values. When the crystallization temperature decreases, the PVDF homopolymer has the lowest nucleation rate values. In most cases, the incorporation of TrFE tends to increase the primary nucleation density and the primary nucleation rate before crystallization starts, according to DSC.

The inverse of the half crystallization time (*τ*_50%_) was experimentally determined as it represents a quantitative measure of the overall crystallization rate that includes both nucleation and growth during the solidification from the melt to the semicrystalline state. During the isothermal crystallization experiments, the half crystallization time is the time needed by the material to attain 50% relative conversion to the semicrystalline state.

**Figure 5:**
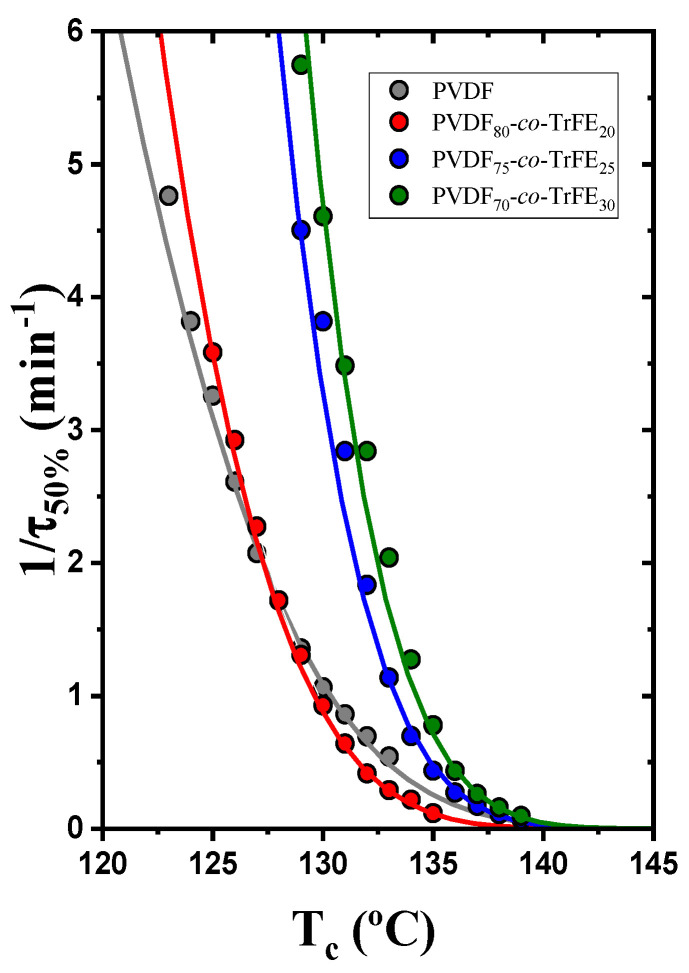
Inverse of the half crystallization time for neat PVDF and copolymers at different compositions as a function of the isothermal crystallization temperature.

Figure 5 shows the inverse of the half crystallization time as a function of the isothermal crystallization temperature. The solid lines plotted were calculated by the Lauritzen and Hoffman theory. The P(VDF_75_-*co*-TrFE_25_) and P(VDF_70_-*co*-TrFE_30_) samples crystallize faster at similar *T_c_* values in comparison to the P(VDF_80_-*co*-TrFE_20_) and neat PVDF, whose overall crystallization rates are similar. A comparison between Figure 5 (where both nucleation and growth influence the results) with Figure 2, where only growth is taken into account, indicates that there is competition between the increase in primary nucleation and the decrease in secondary nucleation (growth) as the TrFE increases in the copolymers. As a result, the increase in primary nucleation seems to be the determining factor in the overall increase in crystallization kinetics for the copolymers with 25 and 30% TrFE in comparison with neat PVDF or the copolymer with the lowest amount of TrFE.

To know the relevance of the growth or the effect of nucleation in the crystallization process, the ratio between the growth rate (*G*) and the inverse of the half crystallization time (1/*τ_50%_*) of the copolymers with respect to the homopolymer is calculated. The results at a selected *T_c_* for both types of measurements are shown in Figure S2. The difference in the ratio is larger in the inverse of the half crystallization time, where the nucleation is taken into account, whereas in the *G* values, only the growth is measured. Therefore, primary nucleation has determining importance in the overall crystallization process of this system.

All the parameters extracted from the fitting of the Lauritzen and Hoffman theory by DSC experiments are listed in Table S2. The equilibrium melting temperature (*T_m_*^0^) values employed in each sample and used in the Lauritzen and Hoffman theory are estimated by the Hoffman–Weeks method (Figure S3 and Table S3) [46,47].

To predict the overall crystallization kinetics during the primary crystallization regime, the Avrami theory was employed. The form of the Avrami equation employed is the following [48]:

(1) $$1-V_{c}\left(t-t_{0}\right)=exp\left(-k{\left(t-t_{0}\right)}^{n}\right),$$

where *V_c_* is the fraction of the relative volume fraction transformed to the semicrystalline state, *t* is the time employed in the experiment, *t*_0_ is the induction time before the crystallization start, *k* is the constant of the overall crystallization rate, and *n* is the Avrami index (related with the time dependence of the nucleation and the crystal geometry).

The Avrami index is composed of two terms [49,50]:

(2) $$n=n_{d}+n_{n},$$

where *n_d_* is the dimensionality of the crystals growing, and *n_n_* represents the nucleation kinetics contribution. For polymers, the dimensionality expected is 2D or 3D, which corresponds to a value of *n_d_* of 2 or 3 for axialitic or spherulitic morphology, respectively. The value of *n_n_* can vary between 0 and 1, where 0 is for instantaneous nucleation, and 1 corresponds to sporadic nucleation.

The application of the Avrami equation in every isothermal experiment allows for obtaining the Avrami index (*n*). To apply this equation, it is necessary that the crystallization process starts when the sample reaches the isothermal crystallization temperature previously selected and not during the cooling step. The *n* value can predict the morphology of the crystals in the isothermal crystallization procedures. If the value is lower than 1.5, the crystals formed are needles (1D). When the value is between 1.5 and 2.4, the crystals should be instantaneously nucleated axialites (2D), and if *n* values are between 2.5 and 3.4, the crystals could be sporadically nucleated axialites or instantaneously nucleated spherulites (i.e., *n* = 3). When the Avrami index is between 3.5 and 4, it is possible to ensure that the crystal morphology is 100% spherulitic (i.e., *n* = 4 for sporadically nucleated spherulites) [48,51,52].

**Figure 6:**
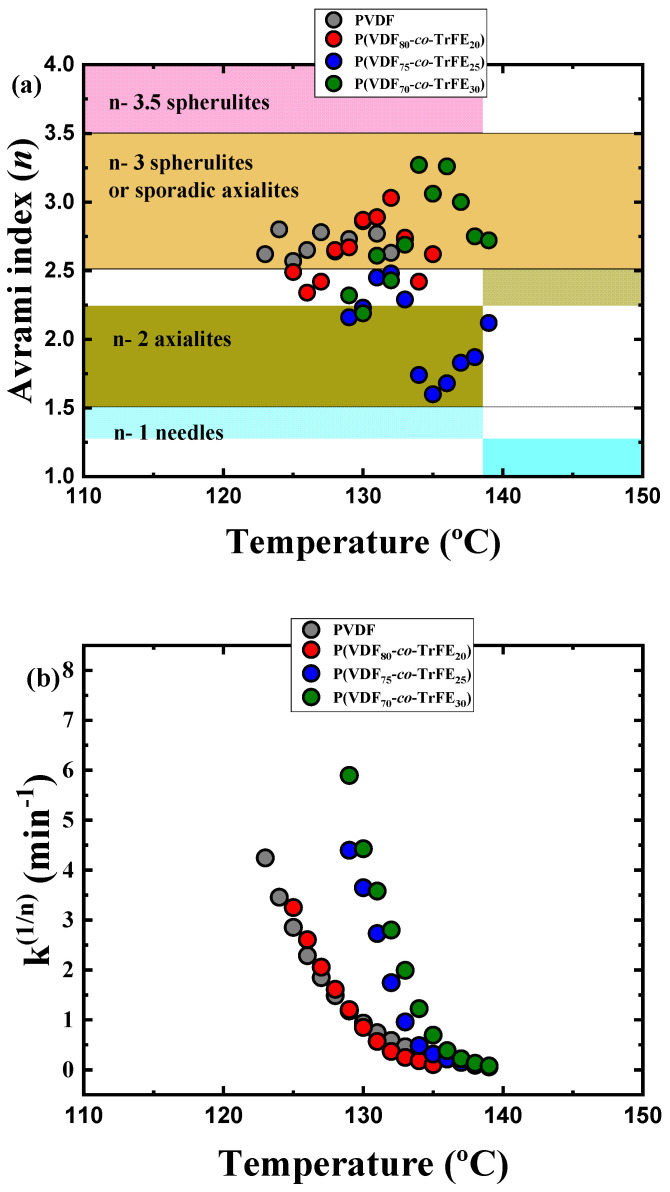
(**a**) Avrami index values for neat PVDF and copolymers at different compositions against their respective isothermal crystallization temperatures and (**b**) crystallization rate obtained by the Avrami model in each isothermal temperature measured.

All the Avrami indexes obtained are presented in Figure 6a. The PVDF homopolymer has all the *n* values higher than 2.5, which is consistent with the spherulitic morphology observed previously by PLOM (Figure 3a). The random copolymers have values between spherulites and axialites, and it is possible to observe how the *n* value decreases when the TrFE content increases. Figure 6a reports the values of the Avrami index close to 2 for the two copolymers with the highest TrFE content that corresponds to instantaneously axialites, which is consistent with the morphologies observed in Figure 3c,d.

Figure 6b plots the *k^(1/n^*^)^ values for each isothermal crystallization temperature. This value is an indication of the overall crystallization rate predicted by the Avrami theory [48,52]. The comparison of these values with those obtained experimentally by DSC (Figure 5) demonstrates the accuracy of the Avrami theory due to the high similarity in all the results gathered.

**Figure 7:**
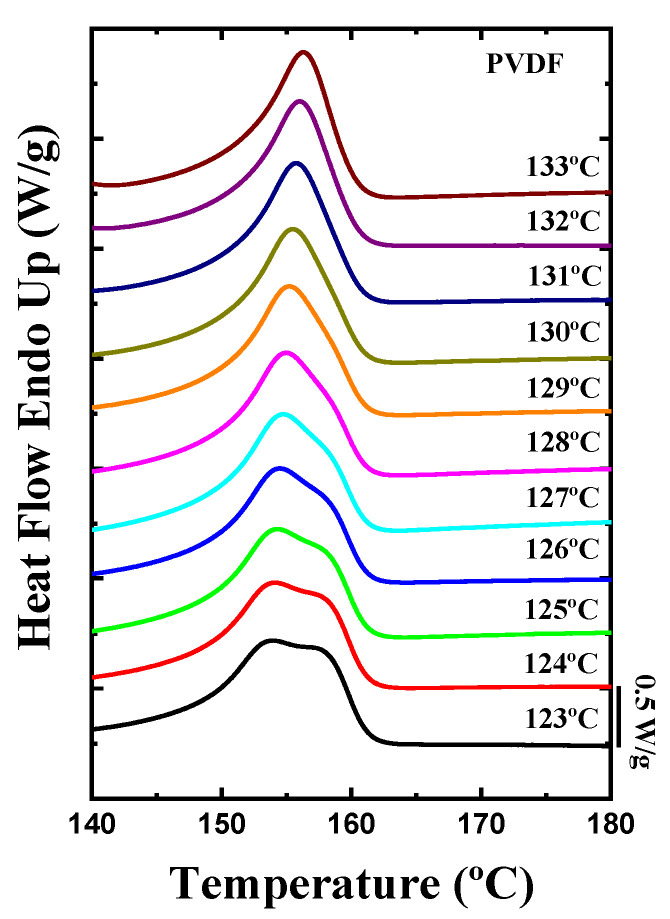
DSC heating scans after the isothermal crystallization process of PVDF homopolymer.

After the isothermal crystallization procedure, analysis of the subsequent DSC heating scans was carried out. Figure 7 presents the DSC heating curves measured immediately after the isothermal crystallization of the PVDF homopolymer sample. In neat PVDF, at low isothermal crystallization temperatures, two melting peaks were observed. The first melting peak, located around 155 °C, corresponds to the α-phase, which is the most common crystalline phase in PVDF when the polymer is crystallized from the molten state [53,54]. The second melting peak (also corresponding to the melting of α-phase crystals) or shoulder observed is the reorganization of the α-crystals during the heating process. This second peak tends to disappear when the isothermal crystallization temperatures increase.

**Figure 8:**
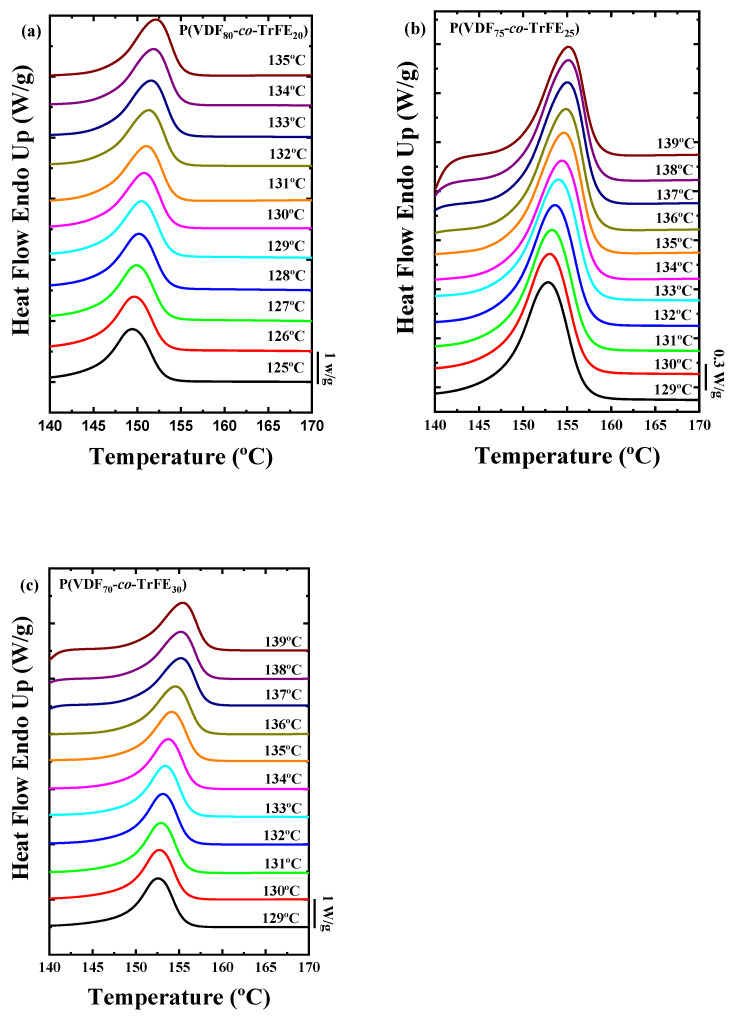
DSC heating curves after the isothermal crystallization process of (**a**) P(VDF_80_-*co*-TrFE_20_), (**b**) P(VDF_75_-*co*-TrFE_25_), and (**c**) P(VDF_70_-*co*-TrFE_30_) samples.

The DSC heating scans for random copolymers at different compositions after the isothermal crystallization processes are presented in Figure 8. All the samples presented have the same behavior, where only one melting peak is observed and located at around 150 °C. This melting peak corresponds to a crystalline structure composed essentially of *TG^+^TG^−^* chains (i.e., the α-phase) because the melting temperature observed occurs at higher temperatures than the Curie temperature for all the samples. All the isothermal crystallization curves for the PVDF homopolymer and the random copolymers are presented in Figure S4.

### 2.3. Self-Nucleation (SN) and Successive Self-Nucleation and Annealing (SSA)

**Figure 9:**
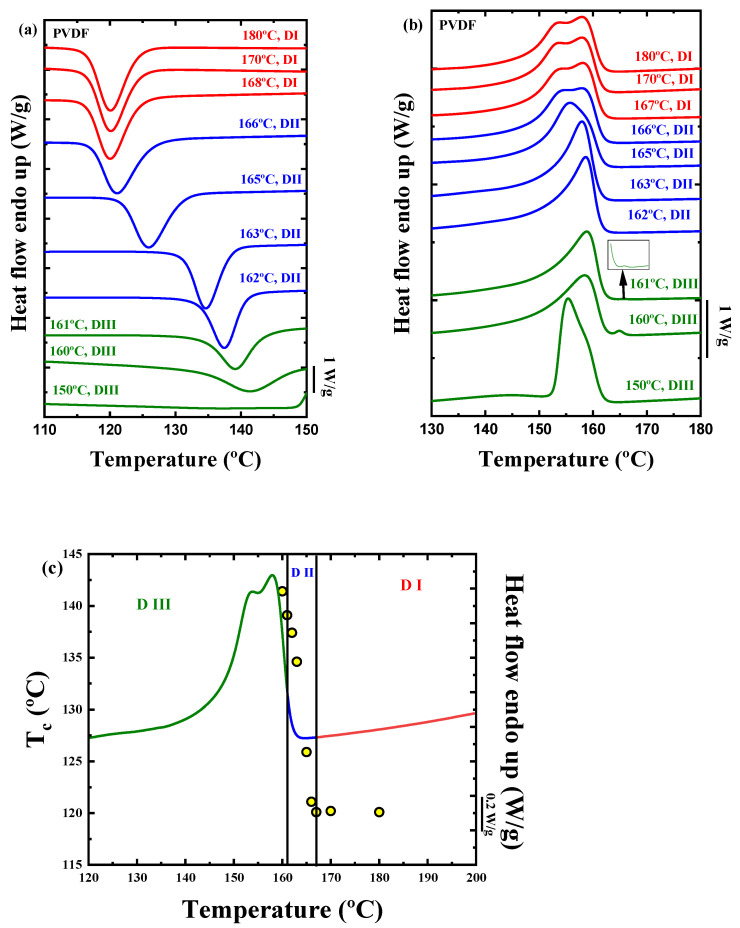
(**a**) DSC cooling scans after 5 min at the indicated *T_s_* values, (**b**) subsequent DSC heating scans for the PVDF homopolymer, and (**c**) representation of each *Domain* in the self-nucleation process superimposed on a standard melting curve of the PVDF homopolymer sample. The circles represent the crystallization temperatures (left *Y*-axis) at the corresponding *T_s_* values (*X*-axis).

In theory, the best nucleating agent for a polymer is made up of its own crystal fragments [55,56,57]. To check the nucleating effect of the TrFE in the PVDF, self-nucleation experiments were carried out in the homopolymer and in the three copolymers studied. Figure 9 shows the results obtained after the SN protocol in the PVDF homopolymer. The cooling scans after the holding time (5 min) at the indicated *T_s_* temperatures are plotted in Figure 9a, and the subsequent heating scans are presented in Figure 9b. The colors of the lines are indicative of the *Domains* where the polymer is, at the temperature indicated. Red denotes *Domain I* (melting *Domain*), blue *Domain II* (self-nucleation *Domain*), and green *Domain III* (self-nucleation and annealing *Domain*). Figure 9c shows the different *Domains* observed superimposed on the standard melting curve of the PVDF homopolymer sample.

In *Domain I*, the melting process of the polymer occurs completely, and the thermal history of the material is erased so that isotropic and relaxed random coils exist in the molten state. For neat PVDF, *Domain I* occurs at temperatures higher or equal to 167 °C (Figure 9), and there are no changes in the crystallization temperature of the material upon cooling from *Domain I*.

*Domain II* encompasses a *T_s_* range where self-nuclei remain in the polymer, but the temperature is not high enough to produce annealing of any unmolten crystal fragments that could act as self-seeds. For more on self-nucleation Domains, the reader is referred to two recent reviews [56,57]. *Domain II* is identified because upon cooling from *T_s_* values located in this *Domain*, the crystallization peak temperature increases as the nucleation density is increased. Finally, *Domain III* occurs when the applied *T_s_* temperature can only partially melt the crystals in the sample, and unmolten crystals anneal (thicken) during the 5 min holding time at *T_s_*; therefore, in the subsequent heating run, an additional melting peak is observed due to the melting of the annealed crystals (Figure 9b).

The PVDF is located in *Domain II* after self-nucleation employing *T_s_* temperatures between 162 °C and 166 °C (see Figure 9a). The lowest *T_s_* value in *Domain II* is known as the ideal self-nucleation temperature, *T_s ideal_*, as it produces the maximum self-nucleation effect (i.e., the maximum increase in *T_c_* values) without any annealing. The nucleation density is increased exponentially as *T_s_* is decreased in *Domain II*. This nucleation density increase produces a shift of the crystallization temperature to higher values. This behavior is observed in Figure 9c when the material is in the range of temperatures within *Domain II*. The increase in the crystallization temperature in *Domain II* can cause small changes in the melting point, as observed in Figure 9b. At 166 °C, the PVDF exhibits a bimodal melting peak as a result of reorganization during the scan. As the *T_s_* temperature is lowered to 163 °C, the melting turns monomodal, as crystallization takes place at much higher temperatures during cooling, already producing more stable crystals that do not reorganize during melting.

The PVDF homopolymer shows a small annealing peak at *T_s_* = 161 °C, signaling the onset of *Domain III* (Figure 9b). From this temperature to lower values of *T_s,_* the material is located in *Domain III*. The self-nucleation behavior of PVDF is typical of most semicrystalline polymers in bulk, displaying the three SN Domains and very clear transitions between them [56,57].

**Figure 10:**
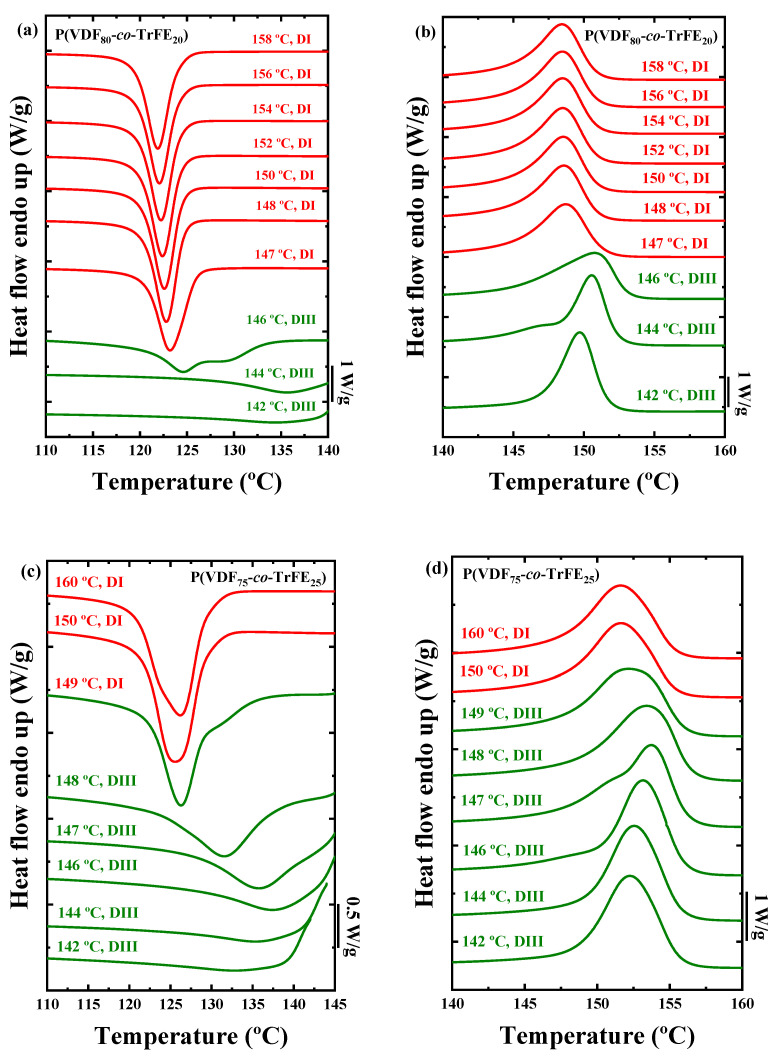

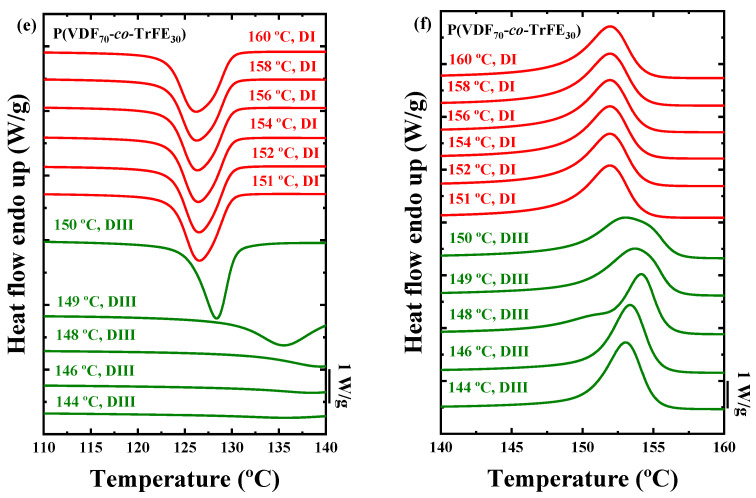
DSC cooling sweeps after 5 min at the indicated *T_s_* values for (**a**) PVDF_80_-*co*-TrFE_20_, (**c**) P(VDF_75_-*co*-TrFE_25_) and (**e**) P(VDF_70_-*co*-TrFE_30_) samples, and DSC heating scans after the cooling process for (**b**) P(VDF_80_-*co*-TrFE_20_), (**d**) P(VDF_75_-*co*-TrFE_25_), and (**f**) P(VDF_70_-*co*-TrFE_30_) samples.

The results obtained by the self-nucleation protocol in the random copolymers are displayed in Figure 10. There is a large difference between the PVDF and the random copolymers. In the three random P(VDF-*co*-TrFE) copolymers, *Domain II* is absent. The TrFE content in the copolymers affects the self-nucleation process, and it can be observed how the *T_s_* value range in each *Domain* is altered with the composition.

The P(VDF_80_-*co*-TrFE_20_) sample is in *Domain I* at *T_s_* values of 147 °C and higher. Upon decreasing the self-nucleation temperature to 146 °C, the material directly transitions to *Domain III*. The P(VDF_75_-*co*-TrFE_25_) sample jumps directly from *Domain I* to *Domain III* at a *T_s_* value of 150 °C. Finally, in sample P(VDF_70_-*co*-TrFE_30_), there is a jump from *Domain I* to *Domain III* at 151 °C, i.e., there is no *Domain II* presence in this sample. To appreciate these jumps between *Domains* better, the crystallization and melting enthalpies against the *T_s_* values are presented in Figure S5, where it is possible to observe how the crystallization enthalpy decreases when the material is in *Domain III*. In addition, the melting temperature and the crystallization temperature of the curves after the self-nucleation protocol at the corresponding *T_s_* values are plotted in Figure S6 in order to better observe the change of the different *Domains* during the experiments. The indicative standard melting curves of the copolymers with *Domains I* and *III* marked in red and green colors, respectively, are plotted in Figure S7 in the Supporting Information section.

As the intrinsic nucleation density in polymeric materials increases, *Domain II* tends to reduce its width and eventually disappears. This behavior is typical of many high-density polyethylenes [58]. In the case of PVDF, the material clearly exhibits the three self-nucleation *Domains*, but when the TrFE counits are incorporated randomly into the copolymers, the nucleation density increases so much that the material is incapable of being self-nucleated without undergoing annealing. As in the case of HDPE, there seems to be a saturation value of the nucleation density above which self-nucleation without annealing is not possible anymore, and *Domain II* disappears. These results are consistent with the morphology change and the reduction of Avrami indexes observed in the copolymers.

**Figure 11:**
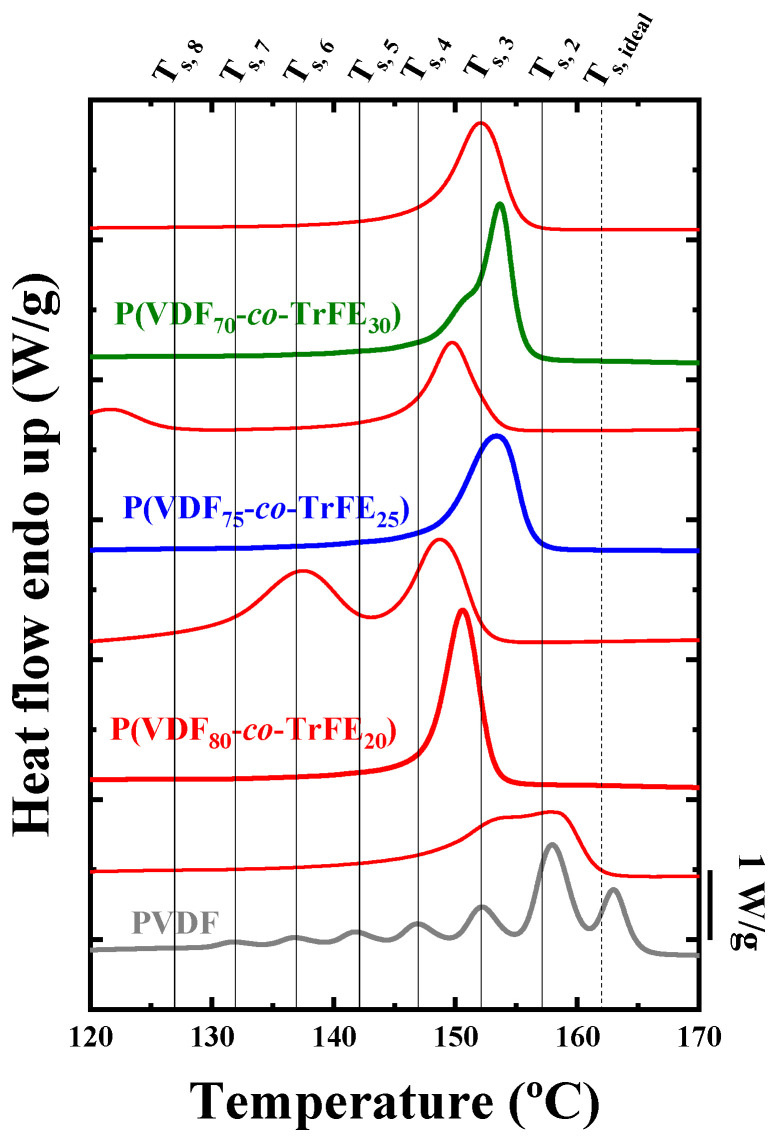
Final DSC heating scans after the SSA protocol for neat PVDF and the indicated copolymers. The name of each sample is written on top of each final SSA DSC scan. For comparison purposes, the DSC scans drawn with thin red lines (above each DSC scan after SSA) correspond to the standard DSC heating scans of the corresponding samples.

The SSA treatment was carried out for all the samples, and the heating curves after the fractionation processes are collected in Figure 11. The vertical lines in the figure indicate the *T_s_* employed for the fractionation of the materials. The heating curve after SSA for the PVDF homopolymer sample reveals that it can be thermally fractionated. The DSC trace shows a series of endothermic peaks representing thermal fractions with different lamellar thicknesses (the higher the *T_m_* value, the thicker the average lamellae). PVDF exhibits a monomodal fractionation profile after SSA that is probably proportional to its molecular weight distribution and/or intermolecular interactions. Linear PVDF should not contain defects that interrupt its crystallizable sequences. However, a small number of head-to-tail additions during polymerization could be present and may also facilitate molecular segregation during crystallization and, hence, thermal fractionation. In perfectly linear polymers without any defects that can interrupt the crystallizable sequences, the two possible sources for fractionation are the distribution of molecular weights [56,59] and the existence of intermolecular interactions capable of acting like sticky “defects” in the chains [60]. This last effect is present in most polar molecules, so its presence in PVDF is also possible.

Unexpectedly, the random copolymers exhibit a very different SSA thermal fractionation profile. For the copolymer with the lowest TrFE incorporation, there is only one melting endotherm after SSA and a small shoulder at lower temperatures, which seems to be an ill-defined second thermal fraction. In any case, the thermal fractionation capacity dramatically decreased in this P(VDF_80_-*co*-TrFE_20_) sample. The other two copolymer samples with a higher amount of TrFE cannot undergo thermal fractionation during the SSA process. It is well known that incorporating comonomers in random copolymers where counit exclusion predominates during crystallization significantly increases the SSA thermal fractionation capacity [56,59]. In the present case, the TrFE comonomer incorporation in the copolymer chains does not lead to an increase in fractionation capacity. Therefore, the results presented in Figure 11 evidence that TrFE countis are included within the PVDF crystals.

Nevertheless, the total lack of fractionation in these P(VDF-*co*-TrFE) random copolymers is unexpected and represents an outstanding result in the field of SSA thermal fractionation. Materials such as HDPE homopolymers that are 100% linear and apolar do not experience fractionation (or the fractionation is very limited), a fact that has been attributed to the low sensitivity of nonpolar HDPE chains to become fractionated based only on molecular weight distribution [51,56,61,62]. What is remarkable about the results presented here is how fractionation not only does not increase with comonomer incorporation, as one would have expected when comonomer exclusion dominates the behavior, but it is strongly inhibited. The SSA protocol in the random P(VDF-*co*-TrFE) copolymers only produces annealing of the samples, thereby increasing their melting temperatures in comparison to the samples crystallized from the melt at 20 °C/min, as shown in the thin red lines extracted from Figure 1b. The total lack of fractionation in the copolymers is difficult to explain as it will depend on the exact origin of the SSA fractionation ability of PVDF [61,62].

### 2.4. Ferroelectric Measurements

**Figure 12:**
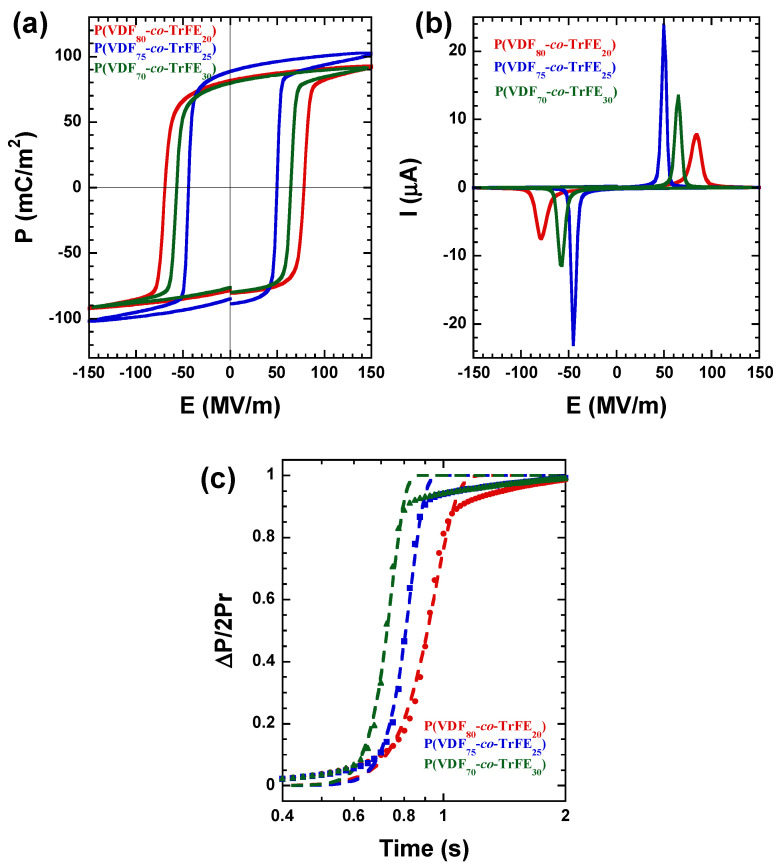
(**a**) Polarization vs. electric field hysteresis loop for the three P(VDF-*co*-TrFE) based compositions studied, (**b**) the corresponding current vs. electric field data, (**c**) switching transients of copolymers as a function of time at room temperature at a constant electric field of 150 MV/m, and dashed lines are the fits according to the KAI model.

Figure 12a presents polarization as a function of electric field (*P* vs. *E*) hysteresis loops obtained by applying an external electric field of 150 MV/m at a frequency of 0.1 Hz for the three different copolymer compositions studied (80/20, 75/25, and 70/30). Considering that the processing conditions were the same for the three of them, the ferroelectric response for the P(VDF_75_-*co*-TrFE_25_) is the best. The remnant polarization, *P_r_*, value is 89 mC/m^2^ for the processing conditions explained before. The other two compositions exhibit lower values of *P_r_*, being 82 mC/m^2^ for the P(VDF_80_-*co*-TrFE_20_) sample and 80 mC/m^2^ for the P(VDF_70_-*co*-TrFE_30_) sample. The coercive field, *E_c_*, value is higher for the P(VDF_80_-*co*-TrFE_20_) sample, 78 MV/m, and is reduced for the other two samples, being 65 MV/m for the P(VDF_70_-*co*-TrFE_30_) composition and even lower for the P(VDF_75_-*co*-TrFE_25_) sample, 50 MV/m. Figure 12b presents the corresponding electric current as a function of the electric field (*I* vs. *E*) curves. Sharper switching peaks are observed for 75/25 and 70/30 compositions, which suggest faster ferroelectric switching. With these results, it is possible to establish that the P(VDF_75_-*co*-TrFE_25_) sample manifests the best ferroelectric response in terms of higher *P_r_*, lower *E_c_*, and a faster switching rate.

To understand the mechanism of polarization switching, the results are considered with the nucleation and growth theory described by a model developed by Ishibashi and Tagaki [63], the so-called Kolmogorov–Avrami–Ishibashi (KAI) model, and based on the classical Kolmogorov [64] and Avrami [65] theory. This model considers that the following equation can describe the switching transient as a function of time:

(3) $$∆P\left(t\right)/2P_{r}=1-\exp\left[-{\left(\frac{t}{t_{0}}\right)}^{n^{\prime }}\right],$$

where *t*_0_ is the characteristic switching time, and *n′* is a parameter proportional to the dimensionality of the polarization switching.

Figure 12c shows a typical polarization transient at room temperature for the different compositions studied. The dashed lines in Figure 12c are the fitting curves in order to indicate that the KAI model can fit the experimental data. The dimensionality of the switching mechanism in ferroelectric polymers is still unclear, and several works have been published during the last few years trying to solve or explain this issue [66,67,68,69]. The study of the dimensionality of the switching mechanism is outside the scope of this work.

The results obtained here indicate that the switching time and coercive field decrease with increasing TrFE content. However, when the TrFE content is increased above a certain point (above 25% in the case of the samples examined here), the maximum and remnant polarization starts to decrease due to the lower dipole moment of the TrFE defects, 1.4 D compared with 2.1 D for VDF, in the crystalline lamellae [70].

## 3. Materials and Methods

### 3.1. Materials

A commercial PVDF is used in this work (Sigma-Aldrich, Munich, Germany), *M*_w_ = 180,000 g/mol, M_n_ = 71,000 g/mol) as a homopolymer. Different random copolymers of P(VDF-*co*-TrFE) with different molar ratios were supplied by Piezotech^®^ FC (Pierre Benite France). In this work, 80/20, 75/25, and 70/30 VDF/TrFE molar ratios were used.

### 3.2. Methods

#### 3.2.1. Differential Scanning Calorimetry (DSC)

A Perkin Elmer DSC 8000 with an Intracooler II as a cooling system was employed to carry out the DSC experiments. The equipment was calibrated with indium and tin standards.

The non-isothermal procedure consists of a first heating scan of the material to 20 °C above the melting temperature and holding the sample at that temperature for 3 min to erase the thermal history. Then, the sample is cooled down at 20 °C/min from the molten state to 25 °C and held for 1 min at this temperature. After this step, a new heating scan at 20 °C/min is performed up to the molten state.

For the isothermal crystallization experiments, the protocol employed was the same described by Müller et al. [48,52]. First, the minimum crystallization temperature (*T_c,min_*) is estimated. To find this temperature, the sample is heated to the molten state (20 °C above the melting temperature) and held for 3 min at this temperature. The following step is cooling the sample at 60 °C/min to a crystallization temperature (*T_c_*) previously selected. When this *T_c_* is reached, the sample is immediately heated up at 20 °C/min to the previously selected melt temperature. If no melting peak is appreciated during this second heating scan, this is a valid crystallization temperature. The experiments are repeated at increasingly lower *T_c_* values until a melting peak is found during the subsequent heating scan, indicating that the sample was able to crystallize during cooling at 60 °C/min. Hence, this temperature is discarded, and the immediately higher *T_c_* value is employed as *T_c,min_*.

Once the value of the *T_c,min_* is obtained, the isothermal crystallization experiments are carried out in the widest possible experimental range. As in the previous experiments, the sample is heated up to 20 °C above the melting temperature and maintained for 3 min at this temperature. Then, the sample is quickly cooled down (60 °C/min) to a previously selected *T_c_* and held at this *T_c_* for 40 min to let the sample crystallize until saturation. When the crystallization process is finished, the sample is heated at 20 °C/min to the molten state. The process starts again with the next *T_c_* selected.

**Figure 13:**
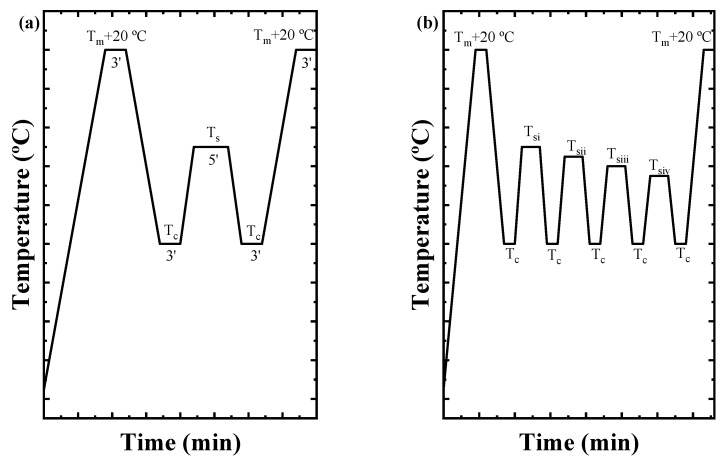
Scheme of all the steps necessary to perform the (**a**) self-nucleation (SN) protocol and (**b**) the SSA thermal fractionation.

The self-nucleation (SN) experiments were performed following the protocol recommended by Müller et al. [56,58]. All the scans carried out during the SN experiments were made at 20 °C/min. First, the thermal history of the material is erased at 20 °C above the melting temperature for 3 min. For the next step, the sample is cooled from the molten state to a low temperature to ensure the crystallization of the material (100 °C for the PVDF homopolymer and random copolymers) and is held for 3 min at this temperature. Then, the sample is heated to a previously selected SN temperature, *T_s_*, and remains at this temperature for 5 min. The following step is cooling down the sample from the *T_s_* to the crystallization temperature chosen and keeping the sample for 3 min at this temperature. In this step, depending on the *Domain* that the sample is in, some changes in the value of *T_c_* can be observed towards higher values in comparison with the previous *T_s_* employed. Finally, the sample is heated again to the molten state, also this step is important to monitor the possible annealing process that occurs in this *Domain* and can be appreciated in the subsequent melting peaks. After this step, the experiment can be repeated by changing the *T_s_* value to another one. Briefly, three *Domains* can be appreciated or distinguished during the SN process for the materials. A material is in *Domain I* when the melting process of the material occurs completely, and the thermal history of the sample is erased. In *Domain II*, the material can self-nucleate, but the temperature is not high enough to provoke annealing. When an annealing peak is detected, the sample is within *Domain III.* In the results and discussion section, in the self-nucleation part, there is an extensive explanation for each *Domain,* and the behavior of the sample in each *Domain* can be appreciated. Figure 13a shows all the steps in the SN protocol.

The Successive Self-nucleation and Annealing (SSA) experiment was carried out following the protocol designed by Müller et al. [59,71]. As in the SN procedure, all the scans were performed at 20 °C/min. The first step is to erase the thermal history of the sample by heating it 20 °C above the melting temperature, keeping the sample at that temperature for 3 min. Then, the sample is cooled down to the same crystallization temperature chosen before in the SN protocol (100 °C in this work for all the samples). After 3 min at that temperature, the sample is heated to the ideal self-nucleation temperature (*T_s,ideal_*) and maintained for 5 min at this temperature. The *T_s,ideal_* is the lowest temperature observed in *Domain II* during the SN experiment. In this work and in order to compare the samples between them, the *T_s,ideal_* chosen for all the samples corresponds to the *T_s,ideal_* obtained for the PVDF homopolymer. During the procedure, the sample is cooled again to the crystallization temperature and held at that temperature for 3 min. This protocol is repeated, decreasing the *T_s_* value to 5 °C compared to the previous cycle in each process. Finally, the sample is heated to the molten state to observe the results of the thermal fractionation. Figure 13b represents the steps to perform the SSA process.

#### 3.2.2. Wide Angle X-ray Scattering (WAXS)

The systems were studied by wide-angle X-ray scattering (WAXS) on a Bruker D8 Advance diffractometer (Bruker, Bremen, Germany) working in parallel beam geometry with Cu K_α_ transition photons of wavelength λ = 1.54 Å. The measurements were performed at room temperature in reflection mode (θ-2θ configuration) after a heating–cooling process to erase the samples’ thermal history, varying the scattering angle 2θ from 10° to 30° with steps of 0.05°. The scattered intensities are shown as a function of momentum transfer Q, Q = 4π λ^−1^ sin θ.

#### 3.2.3. Polarized Light Optical Microscopy (PLOM)

The equipment employed to analyze the samples was an Olympus BX51 polarized optical microscope with a Linkam hot-stage coupled to control the temperature and the heating and cooling rates. To control the thermal process, liquid nitrogen was employed in the Linkam hot-stage. The micrographs were taken by an Olympus SC50 camera linked to the microscope. The samples were previously dissolved in DMF, and the solutions with a concentration of around 4% were drop-casted on a glass substrate and dried at room temperature before the measurements. The growth rate of the spherulites observed was calculated from the slope of the spherulite radius versus time plots, which were always found to be linear.

#### 3.2.4. TF Analyzer

Ferroelectric measurements, basically polarization hysteresis loops, were performed on capacitors and recorded at room temperature using the TF Analyzer 2000E of aixACCT Systems. A continuous sinusoidal wave with a 0.1 Hz frequency was used, and a 150 MV/m electric field was applied to ensure saturation. To prepare the capacitors, the aluminum (Al) electrodes were thermally evaporated onto clean glass substrates to form 100 nm thick bottom electrodes (ME400B PLASSYS evaporator), where the P(VDF-*co*-TrFE) films are later coated. Then, 100 nm thick top Al electrodes were finally thermally evaporated. The temperature inside the evaporator was kept below 70 °C. The sample preparation was performed by taking a solution containing 10 wt% of P(VDF-*co*-TrFE) (for three different compositions) in cyclopentanone and spin-coating it on previously prepared Al/glass substrates. Before the experiments, an annealing process was carried out in all three studied samples. The samples were heated from room temperature to 135 °C, and they were kept at this temperature for 15 min, following the procedure published by Spampinato et al. [38].

## 4. Conclusions

The results obtained in this work are consistent with literature reports that indicate that TrFE units can be included in the PVDF crystal lattice. Such inclusion can decrease the isothermal growth rate of crystals at high crystallization temperatures but, on the other hand, substantially increase the nucleation rate and nucleation density in the copolymers. The increase in nucleation rate dominates the overall crystallization kinetics of the copolymers, provoking an increase in the resulting crystallization rate with respect to neat PVDF.

The remarkable increase in nucleation density provoked by TrFE inclusion in the copolymers causes the disappearance of *Domain II*, as the nucleation density is so high that self-nucleation cannot induce further nucleation. SSA results indicate that the copolymers cannot be fractionated in contrast with neat PVDF. This is consistent with the inclusion of TrFE chains within the PVDF crystal lattice.

Finally, polarization studies have indicated that the P(VDF_75_-*co*-TrFE_25_) sample manifests the best ferroelectric response in terms of higher Pr, lower Ec, and faster switching rate. Above 25% TrFE inclusion in the PVDF crystals, the maximum and remnant polarization starts to decrease due to the lower dipole moment of the TrFE defects.

## Figures and Tables

**Table 1:** Calorimetric data of all the samples obtained after the DSC heating and cooling scans at 20 °C/min.

Sample	*T_c_* (°C)	Δ*H_c_* (J/g)	*T_m_* (°C)	Δ*H_m_* (J/g)	*T_curie, c_* (°C)				*T_curie, h_* (°C)	
PVDF	120	38.4	158	31	-				-	
P(VDF_80_-*co*-TrFE_20_)	120	28.9	149	25.1	83				137	
P(VDF_75_-*co*-TrFE_25_)	124	20.2	150	19.5	74	68		62	112	122
P(VDF_70_-*co*-TrFE_30_)	126	26.3	152	26.1	66		59		105	

## Data Availability

The original data are available from the authors upon request.

## Supplementary Materials

The following supporting information can be downloaded at: https://www.mdpi.com/article/10.3390/ijms231810365/s1.
