# Supplementary material for: Effect of the TrFE Content on the Crystallization and SSA Thermal Fractionation of P(VDF-co-TrFE) Copolymers
Source: Int J Mol Sci. 2022 Sep 8;23(18):10365. doi: 10.3390/ijms231810365 (PMC9499170; doi:10.3390/ijms231810365)
Supplement: Supplementary file 1 [file ijms-23-10365-s001.zip › ijms-1861591-supplementary.pdf]

Effect of the TrFE Content on the Crystallization and SSA thermal fractionation of P(VDF-*co*-TrFE) copolymers

Nicolás María[1], Florian Le Goupil[2], Dario Cavallo[3], Jon Maiz[1,4,5]* and Alejandro J. Müller[1,5]*

[1] POLYMAT and Department of Polymers and Advanced Materials: Physics, Chemistry and Technology, University of the Basque Country UPV/EHU, Paseo Manuel de Lardizábal 3, 20018 Donostia-San Sebastián, Spain.

[2] Laboratoire de Chimie des Polymères Organiques (LCPO - UMR 5629), Bordeaux INP, Universitè de Bordeaux, CNRS, 16 Av. Pey-Berland, 33607 Pessac, France

[3] Department of Chemistry and Industrial Chemistry, University of Genova, 16146 Genova, Italy.

[4] Centro de Física de Materiales (CFM) (CSIC-UPV/EHU)-Materials Physics Center (MPC), Paseo Manuel de Lardizábal 5, 20018 Donostia-San Sebastián, Spain

[5] IKERBASQUE, Basque Foundation for Science, Plaza Euskadi 5, 48009 Bilbao, Spain.

***Corresponding author**: jon.maizs@ehu.eus
alejandrojesus.muller@ehu.es

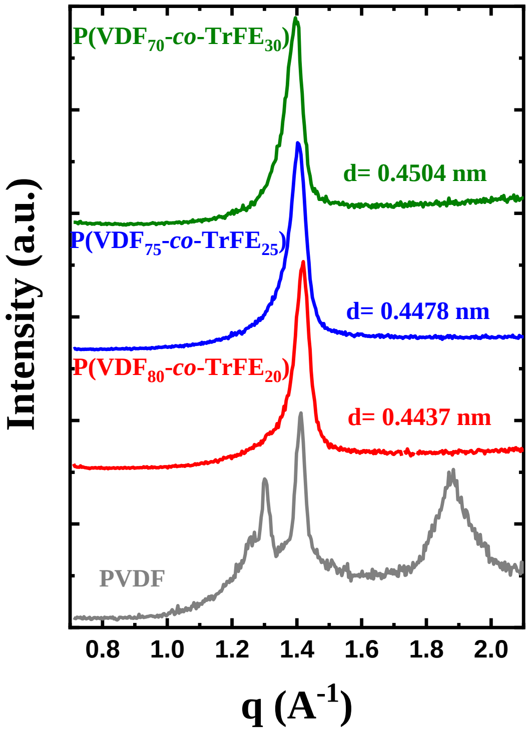

Figure S1. WAXS analysis of the PVDF homopolymer and P(VDF-*co*-TrFE) copolymers after a cooling scan at 20 ºC/min. The *d* values were added in order to appreciate the TrFE inclusion in the PVDF crystals.

Table S1. List of the parameters obtained through the Lauritzen and Hoffman theory applied in the PLOM experiments.

Sample	Kg^G (K^2)	R^2	σ_e (erg/cm^2)	σ(erg/cm^2)	Q (erg)
PVDF homopolymer	6.73x10^4	0.970	60.4	7.86	2.2x10^{-13}
P(VDF$_{80}$-*co*-TrFE$_{20}$)	3.86x10^4	0.998	35.1	7.86	1.29x10^{-13}
P(VDF$_{75}$-*co*-TrFE$_{25}$)	9.70x10^4	0.970	87.5	7.86	3.23x10^{-13}
P(VDF$_{70}$-*co*-TrFE$_{30}$)	5.84x10^4	0.970	52.4	7.86	1.93x10^{-13}

The values of K_g^G observed in Table S1 are the parameters obtained from Figure 3. These values are valid for the overall crystallization temperatures as the L-H solid lines exhibit in Figure 3. In the case of the neat PVDF, at high temperatures the *G* values are higher than the copolymer values, however when the isothermal temperature decreases the *G* values of the neat PVDF decreases and tends to lower values. Therefore, the K_g^G values of the samples do not have any tendency with the proportion of TrFE or PVDF, because the overall values are compensated for the whole range of temperatures.

Table S2. All the parameters calculated through the Lauritzen and Hoffman theory applied for every sample by DSC experiments.

Sample	K_g^τ (K^2)	R^2	σ_e (erg/cm^2)	σ (erg/cm^2)	q (erg)
PVDF homopolymer	1.29x10^5	0.996	116.2	7.86	4.29x10^{-13}
P(VDF$_{80}$-*co*-TrFE$_{20}$)	1.26x10^5	0.998	113.9	7.86	4.20x10^{-13}
P(VDF$_{75}$-*co*-TrFE$_{25}$)	1.46x10^5	0.994	131.8	7.86	4.86x10^{-13}
P(VDF$_{70}$-*co*-TrFE$_{30}$)	1.63x10^5	0.995	146.7	7.86	5.42x10^{-13}

In this case the K_g^τ values have a logical tendency with the composition of the copolymer samples. The neat PVDF and P(VDF$_{80}$-*co*-TrFE$_{20}$) samples have similar values as can be observed in Figure 6a. When the content of TrFE increases the values of K_g^τ also increases. In addition, the values of K_g^τ are higher than the values of K_g^G, these

results are consistent because the values of K_g^G only includes the growth of the crystals whereas the K_g^τ includes growth and nucleation processes.

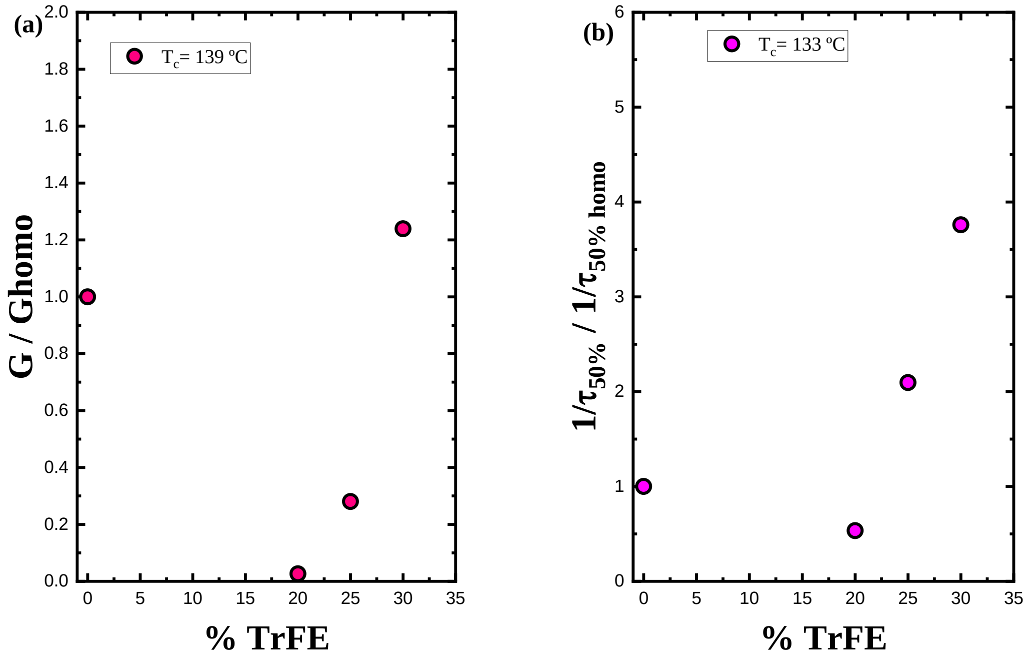

Figure S2. (a) $G/G_{homopolymer}$ relation against the TrFE content at T_c= 139 ºC, and (b) $(1/\tau_{50\%})/(1/\tau_{50\% homopolymer})$ relation against the TrFE content at T_c= 133 ºC.

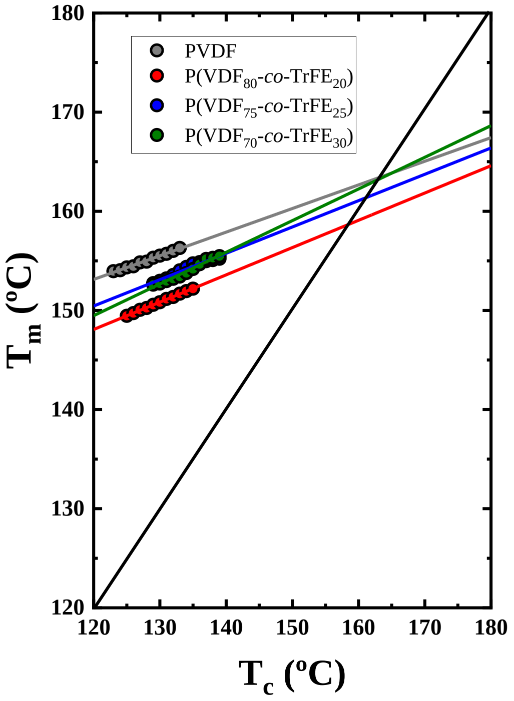

Figure S3. Isothermal crystallization temperature against the melting temperature in order to achieve the equilibrium melting temperature (T_m^0) by Hoffman-Weeks method for neat PVDF and the copolymers.

Table S3. Equilibrium melting temperature values for each sample obtained by Hoffman-Weeks theory.

Sample	PVDF	P(VDF$_{80}$-*co*-TrFE$_{20}$)	P(VDF$_{75}$-*co*-TrFE$_{25}$)	P(VDF$_{70}$-*co*-TrFE$_{30}$)
T_m^0 (ºC)	163.3	158.4	161.1	163.0

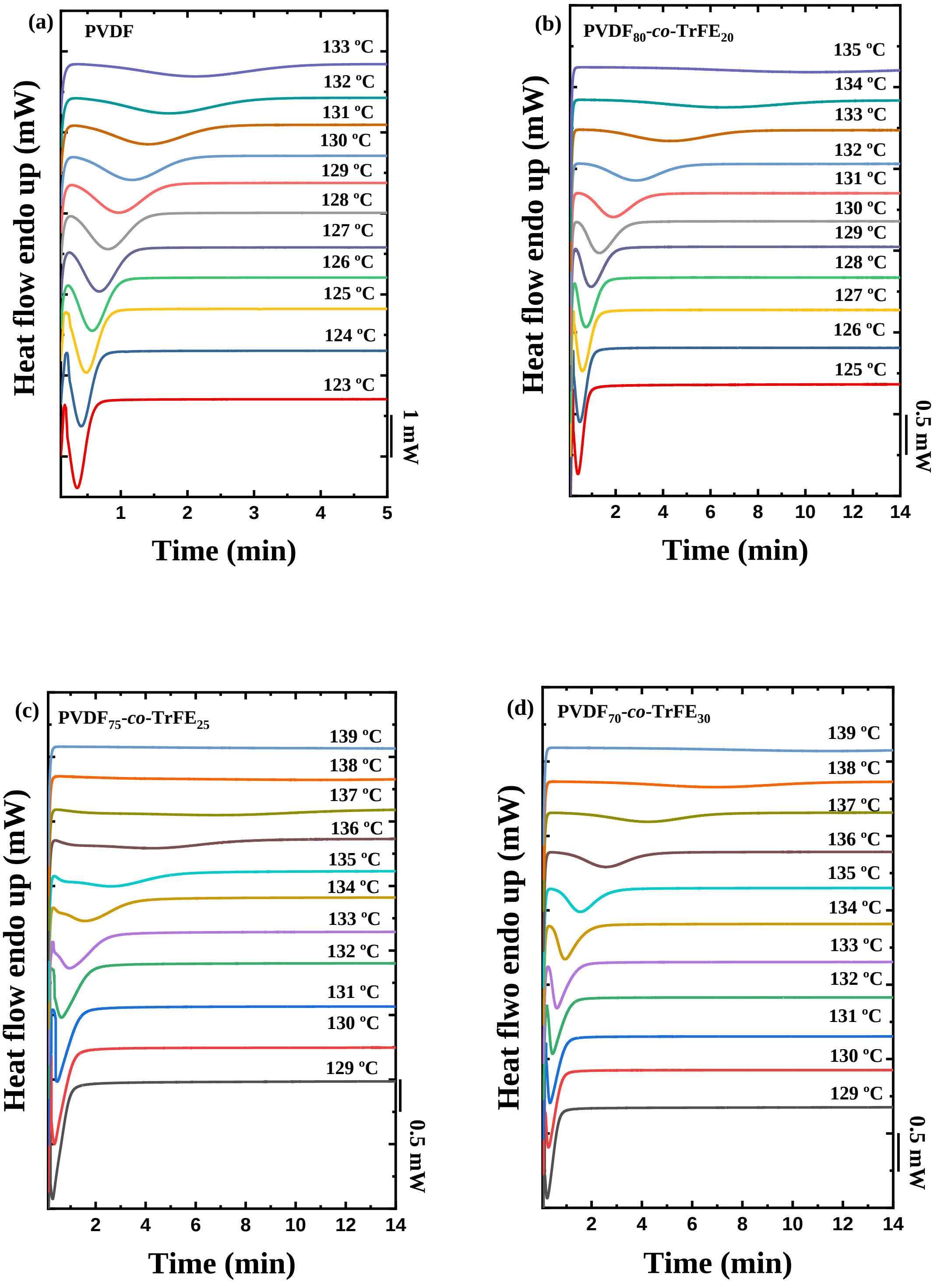

Figure S4. DSC curves of all the isothermal crystallization experiments of (a) PVDF homopolymer, (b) P(VDF_{80}-*co*-$TrFE_{20}$), (c) P(VDF_{75}-*co*-$TrFE_{25}$) and (d) P(VDF_{70}-*co*-$TrFE_{30}$).

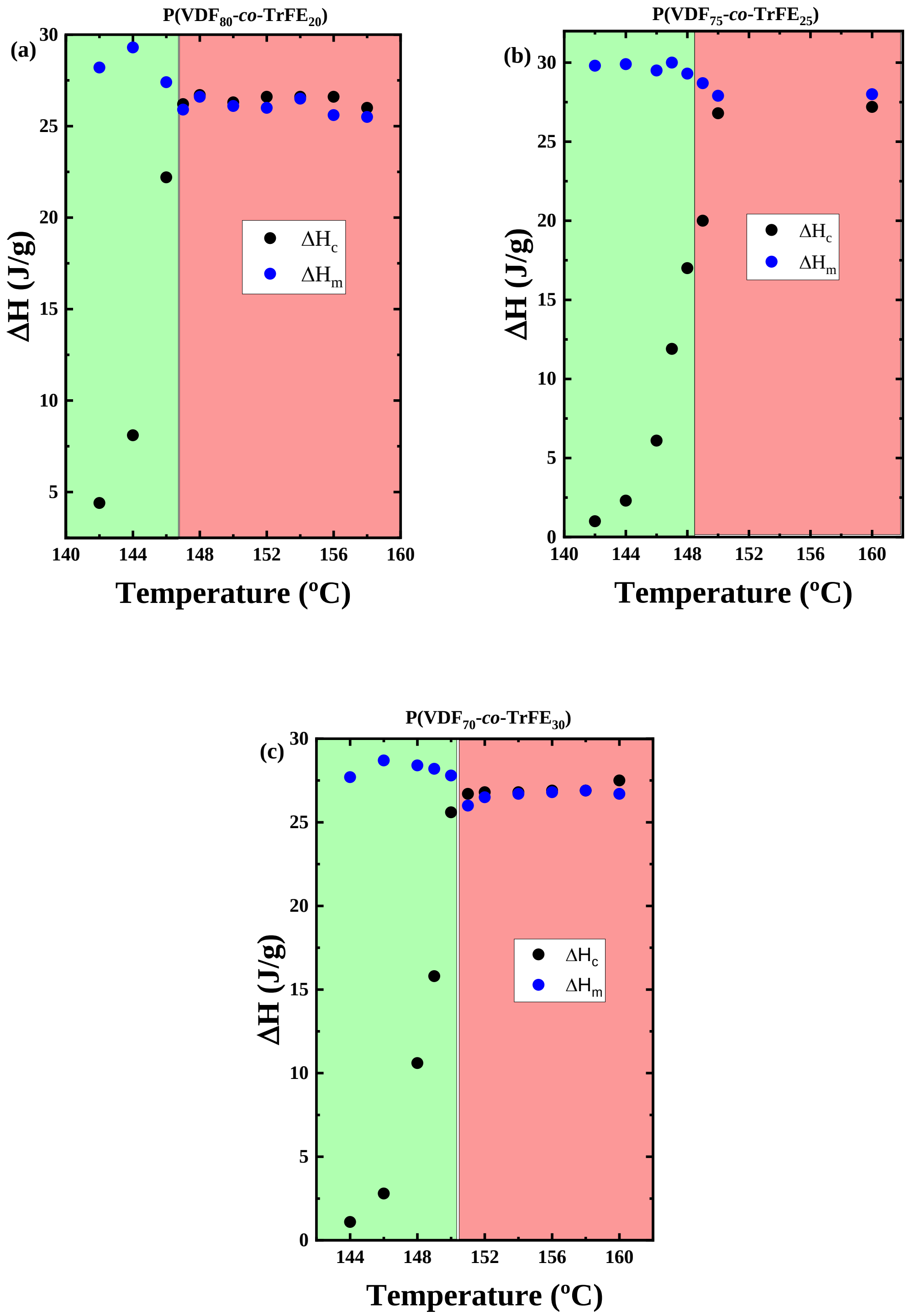

Figure S5. Crystallization and melting enthalpies in each T_s value for the (a) P(VDF$_{80}$-*co*-TrFE$_{20}$), (b) P(VDF$_{75}$-*co*-TrFE$_{25}$) and (c) P(VDF$_{70}$-*co*-TrFE$_{30}$). The green part indicates the temperatures that belong to the *Domain* III and the red part corresponds to the *Domain* I.

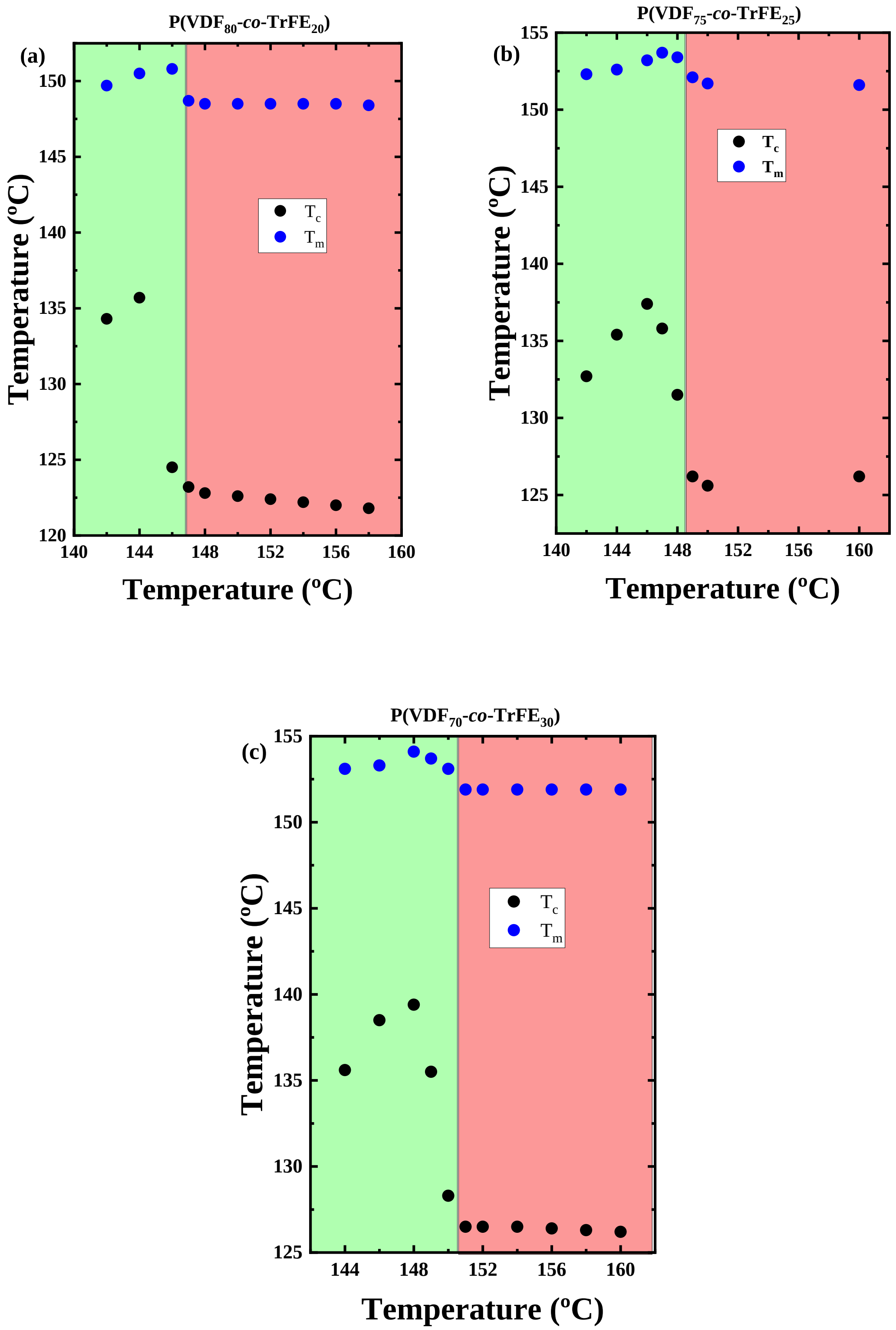

Figure S6. Melting and crystallization temperatures at respective T_s values for (a) P(VDF_{80}-*co*-$TrFE_{20}$), (b) P(VDF_{75}-*co*-$TrFE_{25}$) and (c) P(VDF_{70}-*co*-$TrFE_{30}$). The green part indicates the temperatures that belong to the *Domain* III and the red part corresponds to the *Domain* I.

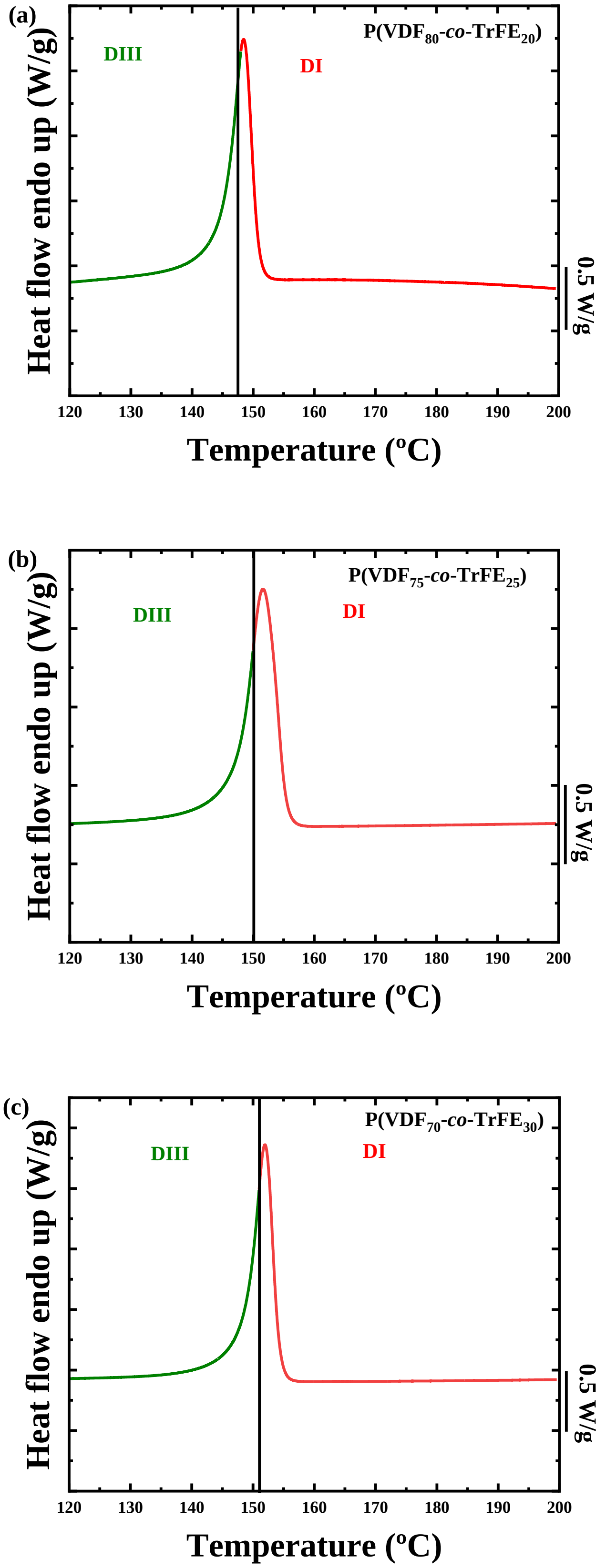

Figure S7. Representation of each *Domain* in the self-nucleation process in a standard melting curve of (a) P(VDF$_{80}$-*co*-TrFE$_{20}$), (b) P(VDF$_{75}$-*co*-TrFE$_{25}$) and (c) P(VDF$_{70}$-*co*-TrFE$_{30}$) samples.
